# The Validity of External:Internal Training Load Ratios in Rested and Fatigued Soccer Players

**DOI:** 10.3390/sports6020044

**Published:** 2018-05-19

**Authors:** Ibrahim Akubat, Steve Barrett, Manuel Lapuente Sagarra, Grant Abt

**Affiliations:** 1 Sport Exercise and Health Research Centre, Newman University, Birmingham B323NT, UK; 2 Sport Medicine and Science Department, Hull City FC, Kingston Upon Hull HU164HB, UK; steve.barrett@hulltigers.com; 3 Spanish Track & Field Royal Association (RFEA), 28008 Madrid, Spain; lapuente.manuel@gmail.com; 4 Development & Innovation of Conditioning & Exercise (DICE) Research Group, INEFC Lleida, 25192 Lleida, Spain; 5 Faculty of Sports Science, European University of Madrid, 28670 Madrid, Spain; 6 Department of Sport, Health and Exercise Science, The University of Hull, Kingston Upon Hull HU67RX, UK; g.abt@hull.ac.uk

**Keywords:** fitness, heart rate, TRIMP, GPS (Global Positionining Systems), PlayerLoad, metabolic power, team sports, monitoring

## Abstract

Purpose: To examine the relationship of external:internal training load ratios with fitness and assess the impact of fatigue. Method: Ten soccer players performed a lactate threshold test followed by two soccer simulations (BEAST_90mod_) 48 h apart. Recovery (TQR) and muscle soreness (DOMS) was measured before each trial. Internal Training load (TL) (iTRIMP) and external load total distance (TD), high intensity distance (HID), PlayerLoad™ (PL) mean metabolic power (MMP) high metabolic power distance (HP) were collected for each trial and external:internal ratios produced. The relationships between ratios and velocity at lactate threshold (vLT) and velocity at Onset of Blood Lactate Accumulation (vOBLA) were examined in both trials along with changes in ratios. Results: Total Quality of Recovery and DOMS showed large changes. There were trivial to large decreases in TL from trial 1 to 2. Moderate increases in ratios for TD:iTRIMP, PL:iTRIMP and MMP:iTRIMP were seen but only small/trivial for HP:iTRIMP and HID:iTRIMP. In rested conditions all ratios show large relationships with vLT and vOBLA. However vLT vs. HID:iTRIMP; PL:iTRIMP; HP:iTRIMP and vOBLA vs. TD:iTRIMP; PL:iTRIMP; MMP:iTRIMP became weaker under fatigue. Conclusions: Acute changes in the ratios have implications forthe use of ratios as fitness measures but also as indicators of fatigue.

## 1. Introduction

The frequent assessment of fitness is important in sports to determine performance capability. However frequent fitness testing in sports with high competition density such as soccer is difficult due to the conflicting requirements and time demands of technical and physical training [1]. There is also the scarcity of periods where fatigue is not a factor in preventing players demonstrating their maximal performance capability during testing. Furthermore, performance measures of physical output are also prone to high variability in game-play making meaningful inferences difficult [2]. One recent development that may be used for the frequent assessment of fitness is the use of an integrated external:internal training load ratio. Akubat, Barrett and Abt [3] demonstrated that combining the individualized training impulse (iTRIMP) with the total distance (TD) and high intensity distance (HID) covered during intermittent exercise showed large relationships with sub-maximal lactate thresholds usually associated with endurance performance capability [4].

The measurement of internal load has long been of interest to sport scientists because it is believed to be the main proponent of the training outcome [5]. Recent research has shown dose-response relationships between measures of internal load (iTRIMP) and changes in fitness in soccer players [6,7]. While the measurement of internal load may be considered preferable, the measurement of external load has become more prevalent and accurate in recent years with the advent and widespread use of Micromechanical Electrical Systems (MEMS) with the integration of GPS technology [8]. The technology has advanced the area of monitoring external physical performance beyond distance and speeds (as was the case with semi-automated camera systems) to include accelerometer-based derivatives of external load (e.g., PlayerLoad™) and more recently the application of the calculations of metabolic power based on the work of numerous authors [9,10,11]. Traditionally, the HID has been used as an external load metric, due to its ability to discriminate between levels of play [12] and the relationship of high intensity activity with changes in fitness [13]. However, some authors have questioned the use of HID because it disregards energetically demanding changes in running speed and it is highly variable between team sports matches [2,11]. An improvement to this [9,10,11,14] has been proposed using the metabolic power model in an attempt to quantify the metabolic cost of acceleration and deceleration [10,11,12,15]. This has been adopted to facilitate and redefine the interpretation of external loads incurred in team sports matches, especially high intensity movements. Metabolic power appears to be a potential solution to the problem of using purely speed-based distance measurements where high-intensity movements are only categorised as such when certain speed thresholds are breached. The limited sprint distance in a soccer game is unlikely to present regular opportunities for high speeds to be reached [10]. Therefore, using speed based thresholds alone does not account for high-intensity movement when the absolute speed threshold is not reached but accelerations take place which could be equally if not more energetically demanding. This concept has been explored by Gaudino et al. [10] who showed that high intensity activity was underestimated by up to 85% in soccer players when using speed thresholds as opposed to the metabolic power model [10]. The metabolic power approach is constrained with compromised measurement accuracy in tracking high acceleration and decelerations with GPS [15] and the models inability to quantify other taxing activities such as impacts, jumps, and changes of direction, which are inherent features of team-sports and are also considered high intensity activity. High-resolution tri-axial accelerometers have also have been incorporated within MEMS (Microelectromechanical Systems) devices. A vector-magnitude algorithm termed PlayerLoad^TM^ (PL) one of the most commonly used metrics in the research literature that is derived from accelerometer data [8,16]. By incorporating movements in all 3 planes of motion into a single measurement PL proposes the advantage of being able to better represent energetic movements in sports than a simple two dimensional model offered by distance measurements or metrics incorporating velocity measurement in just two planes of motion.

Much of the research has described or evaluated external or internal training load metrics in isolation [8]. There is now widespread use of the above-mentioned external load metrics. The previous research demonstrating the potential utility of an integrated external:internal training ratio was limited to use of TD and HID as external load metrics. Therefore the first aim of this study is to re-examine the external:internal load ratio’s with metabolic power and PlayerLoad ^TM^.

We previously mentioned that it is often difficult to assess fitness in season regularly due to high volume of training and match play leaving a scarcity of time where players are free from fatigue. Although the study by Akubat et al. [3] demonstrated that the TD:iTRIMP and HID:iTRIMP ratios showed large relationships with measures of aerobic fitness in rested conditions, research to date has not examined whether these relationships hold true under fatigue or in conditions where players have not fully recovered from the previous exercise stimulus. This is a scenario that is likely to present itself to practitioners. Therefore, the second aim of this paper is to examine if the integrated external:internal ratios relationships with fitness measures hold true when players are not fully recovered and the nature of any change.

## 2. Materials and Methods

### 2.1. Participants

Ten competitive amateur soccer players (Age: 20 ± 1 years; Height: 1.79 ± 0.08 m; Body Mass: 69.87 ± 8.32 kg; VO_2max_: 58.32 ± 5.77 mL·kg·min^−1^) who competed for at least one soccer team were recruited for this study. They provided written informed consent after having all procedures explained to them. The study was approved by the departmental ethics committee at the University of Hull and conformed to the declaration of Helsinki.

### 2.2. Study Design

The study was conducted over a two-week period in the English summer time (June). Participants were asked to avoid any strenuous exercise during the two-week period. During the 1st week a lactate threshold test (LT) was conducted. The LT consisted of six, four minutes stages (6, 8, 10, 12, 14 and 16 km·h^−1^) followed by a ramp to exhaustion which increased at a rate of 0.2 km·h^−1^ every 12 s until the participant could no longer continue. Treadmill gradient was set at 1% for the entire test to reflect the energetic cost of outdoor running [17]. Each stage was separated by one a one minute rest period during which a fingertip capillary blood sample was collected and immediately analysed for lactate (YSI 2300 Stat, YSI Inc., Yellow Springs, OH, USA) in duplicate with the mean being used as the result. Velocity at 2 mmol·L^−1^ (vLT) and 4 mmol·L^−1^ (vOBLA) blood lactate (Bla) were recorded as aerobic fitness measures. Maximal oxygen uptake (VO_2max_) was measured using a breath-by-breath gas analyser (Cortex Metalyzer 3B, Cortex Biophysic, Leipzig, Germany) that was calibrated before and after each test according to the manufacturers’ instructions. VO_2max_ was recorded as the highest mean VO_2_ obtained for a 1 min period during the test [18]. At least two of the following criteria were also required for the attainment of VO_2max_: a plateau in VO_2_ despite increasing treadmill speed, respiratory exchange ratio >1.15, or the attainment of age predicted maximum heart rate (HR_max_) [18]. Heart rate (HR) was recorded using HR belts (Polar Team System, Polar Electro, OY, Kempele, Finland). Mean HR during the last minute of each stage was used in the calculation of the individualised HR-Bla relationship for the calculation of iTRIMP weightings. The peak HR obtained during the test was considered the participants HR_max_.

**Figure 1:**
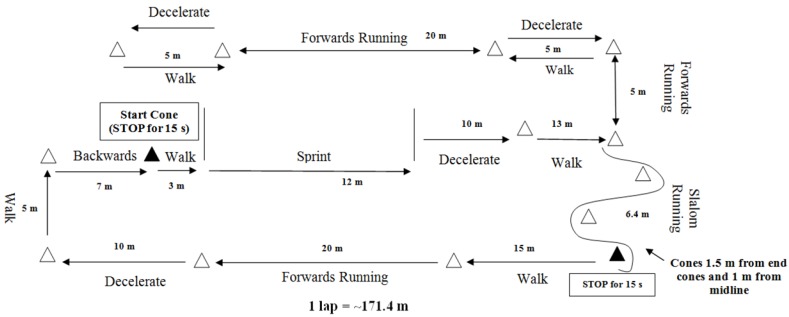
BEAST_90mod_.

Participants were also familiarised with a modified version of the Ball–Sport Endurance and Sprint Test (BEAST_90mod_) adapted from Williams et al. [19] for 30 min (Figure 1). This protocol was chosen for its external validity and reliability in the face of high between match variability from actual match-play [2]. The BEAST_90mod_ has been previously shown to show good reliability (CV 1.7%) [3]. The BEAST_90mod_ is shown in Figure 1. The participants start from the black triangle on Figure 1 (start cone) and perform the different categories of exercise. They walk into a sprint and decelerate into a walk before turning left (up on Figure 1). They carry on to perform the different categories of exercise turning back on themselves and reaching the slalom. After finishing the slalom, they are required to rest for 15 s. The 15 s began as soon as they finish the slalom and end when a research assistant who was timing signified the end of the 15 s. Thereafter the participants would walk and finish the rest of the course arriving at the start cone and again resting for 15 s before starting again.

During week 2 and at least 5 days after the LT test, the participants were asked to perform the BEAST_90mod_ twice on the Wednesday and Friday at the exact same time of day. Participants were provided with a standardized diet for the 48 h before the BEAST_90mod_ and between the two simulations. This standardized diet provided at least 6 g·kg·bm^−1^ carbohydrate, 1.2 g·kg·bm^−1^ protein and 1.5 g·kg·bm^−1^ fat [20]. The diet consisted of a range of foods including milk, yogurt, fruit, cereal, chicken, rice, bread, pasta, fish and vegetables to ensure an adequate intake of macro and micro-nutrients. During the BEAST_90mod_ all players were given 500 mL of water at half time. Before each trial total quality of recovery (TQR) and muscle soreness (DOMS) were subjectively measured using scales previously used [21]. A standardized 15 min warm up consisting of cycling at 50 w for 10 min followed by 5 min of the BEAST_90mod_ was undertaken by all participants. The first participant started the BEAST_90mod_ at 1030 h and the last finished by 1415 h. This time period was selected to minimise any impact circadian rhythms may have on performance. HR data was collected using recordable HR monitors (Polar Team System, Polar Electro, OY, Kempele, Finland) and iTRIMP calculated in a spreadsheet using methods previously described [6,7]. Briefly, iTRIMP is calculated by multiplying the duration exercise (minutes) by the intensity (% heart rate reserve) which is then weighted according to the individual’s exponential heart rate—blood lactate profile. Each recorded HR reading (every 5 s) from the simulation was weighted according to the individual profiles and summated to produce an iTRIMP score. Data for the external load measurement were collected using a 5 Hz GPS system (Minimax, Catapult Innovations, Melbourne, Australia). This provided TD, HID (>15 km·h^−1^), PlayerLoad™ which was provided through dedicated software (Sprint 7.1, Catapult Innovations, Australia). It also provided data for accelerations and velocity which was used in the calculation of mean metabolic power (MMP) and distance covered at high metabolic power (HP; >20 W·kg^−1^). This was calculated using a modified algorithm according to the methods proposed by di Prampero et al. [9], in which accelerated and decelerated running on flat terrain is considered mechanically equivalent to uphill and downhill running at constant speed, and based on the energy cost of constant walking and running depending on hill slope [14], that was later modified by Osgnach et al. [11] for its specific use in football monitoring. The calculation for MMP involved two basic modifications; firstly, the inclusion of the energy spent against air resistance that increases with the square of the speed, as described by di Prampero et al. [9] Secondly, the modification of the algorithm so that when acceleration is below 0, which has been proposed as an energy cost, a “best fit” 4th degree polynomial curve between the acceleration values of 0 and −4.5 m·s^−2^ was used. This last modification alters the value of energy cost obtained by the original algorithm, in which energy cost falls drastically when acceleration is below −4.5 m·s^−2^, and proposes a progressive rise of energy cost when deceleration shows a higher value, corresponding with data from Minetti et al. [14].

### 2.3. Data Analyses

The results were reported as means ± standard deviation. Pearson’s correlation coefficients were used to assess the relationships between external:internal load ratios and aerobic fitness measures. The correlations and qualitative interpretations of the correlation coefficients as defined by Hopkins et al. [22] (0–0.09, trivial; 0.1–0.29, small; 0.3–0.49, moderate; 0.5–0.69, large; 0.7–0.89, very large; 0.9–0.99 nearly perfect; 1 perfect) were used for all correlations along with the 90% confidence intervals (CI) and the associated mechanistic inferences as described by Hopkins et al. [22] The differences in the ratios, recovery measures and training load between the 1st and 2nd BEAST_90mod_ were analysed using standardized effect sizes (Cohens *d*), the qualitative interpretations of the effect size and the 90% confidence intervals of the effect size. The associated mechanistic inferences for the changes were also calculated based on the likelihood of the change all calculated using a bespoke spreadsheet [22,23], *p* values for the comparisons were also reported.

## 3. Results

Table 1 shows the descriptive data of all the load measurements from both simulations. The results showed decreases in all measures apart from MMP and PL where the change was considered unclear. This was accompanied by large increases in DOMS (*d* = 0.84; Large; 0.4 vs. 3.4) and decreases in TQR (*d* = 0.86; Large; 19.7 vs. 13.6) demonstrating that players were not recovered from the first BEAST_90mod_ and the small to moderate reduction in performance (TD and HID) could be due to a lack of recovery and fatigue.

Table 2 shows the changes in the ratios from first BEAST_90mod_ to the second. There were moderate changes in ratios for TD, PL and MMP. Whereas those TL measures only pertaining to part of the simulation (i.e., HP and HID) showed only trivial to small changes and the changes was unclear.

Table 3 shows the relationship between the ratio’s and fitness measures in both simulations. In rested conditions all ratios display a large relationship with vLT and vOBLA. However, these relationships do not always remain the same under fatigued conditions. Some relationships (HID:iTRIMP, PL:iTRIMP, HP:iTRIMP) became weaker and the effect size description changes. This is exemplified by HID:iTRIMP, PL:iTRIMP and HP:iTRIMP where the relationships with vLT changes from “Large” to “Moderate” or “Small” respectively and the relationship becomes unclear in the case of HID:iTRIMP and HP:iTRIMP Similar results are evident with respect to the relationship with vOBLA where relationships for TD:iTRIMP, PL:iTRIMP and MMP:iTRIMP change from “Large” to “Moderate” or “Small” respectively.

## 4. Discussion

The first aim of this study was to assess if more recent MEMS derived metrics such as PL and MP demonstrated the same validity with respect to its relationships with aerobic fitness when used as a ratio with internal load as a previous study [3]. The results showed that in rested conditions all of the external:intenal training load ratios show large relationships with measures of fitness (*r* = 0.58–0.69; Table 3). The largest relationship was 0.69 (TD:iTRIMP) for vLT and 0.67 (HP:iTRIMP) for vOBLA. Given that researchers have claimed the superiority of metabolic power and accelerometry derived external load over distance based measures the results of this study suggests that for analysis of fitness from ratios they all show similar (large) relationships. However, this may differ in actual games as opposed to simulated games where there is a greater stimulus driven energy expenditure which may result in different movement demands especially with accelerations, decelerations, jumps and tackles which could affect both PL and metabolic power. Therefore, these results should be treated with a view to researching the fitness vs ratio relationships in different settings. The similar results also show that using a simple measurement of distance in a ratio would allow fitness measurement. Therefore, those who do not have access to MEMS technology to measure movements, simple field tests or treadmill based tests where the course or protocol distance is known could be used. Such protocols could include circuits like the BEAST_90_ [3,20] where the distance covered could be reasonably measured or even intermittent treadmill protocols [24].

Given the reliable nature of the BEAST_90mod_ (CV < 2%) we can reasonably assume that the moderate reductions in TD, HID, PL and HP signify a decrement in external output due to fatigue. However, the change in MMP was unclear. These results could be the first to demonstrate that MMP may not represent a decrement or fatigue whereas other measures of external load do and this requires further investigation. The results of this study also demonstrate that where examination of external load alone may not help in differentiating between a voluntary reduction in output and an involuntary reduction where the required output is unable to be met a ratio of external:internal training load potentially could provide different information. MMP does not change but when examined as a ratio, the MMP:iTRIMP ratio shows similar changes to TD:iTRIMP and PL:iTRIMP. Therefore, in scenarios such as a game where stimulus driven energy expenditure leads to variation in external load and hence the performance measures cannot be used to suitably inform a decision on decrements in performance, ratios may provide a useful alternative to elucidate if the decrement in external load measures is voluntary or involuntary.

The second aim of this study was to examine if the nature of the relationships between fitness and the ratio’s changed as a result of any fatigue accrued. The results showed that under conditions where players have not fully recovered there were moderate changes in some ratios as shown in Table 2 (TD:iTRIMP, PL:iTRIMP and MMP:iTRIMP) and relationships with fitness became weaker (Table 3). HP:iTRIMP and HID:iTRIMP demonstrate such changes. Therefore, the use of some ratios in scenarios where players are not fully recovered should be treated with caution but also opens an avenue for these ratios potentially to be considered as a detector of fatigue. Although some of the relationships still hold true under fatigue the absolute values change. The relationship between vLT and TD:iTRIMP are similar (*r* = 0.60 and 0.69) in rested and fatigued states, however there was a moderate change in the ratio (30.10 vs. 37.71). Therefore, despite the relationship being maintained, the change in the absolute value of the ratio would imply change (improvement) in fitness. However, we can reasonably assume given the study design that this change cannot be due to any change in fitness. Therefore, such ratios should be used with caution in fatigued states to assess fitness. These characteristics are displayed by HID:iTRIMP and HP:iTRIMP with vOBLA. An increase in the external:internal TL ratios in normal circumstances would normally imply an increase in fitness. However, this is not the case as the increase in the ratios seems to be modulated by the decrease in iTRIMP being greater than the decrease in external load even though both decreased (Table 1). There could be a greater utilization of energy production pathways that do not require oxygen thereby reducing the HR response. Alternatively, this could partly be explained by the suppressed HR response in over-reached athletes [25]. If these are the mechanisms through which such changes in the ratios are seen then the ratios could also be considered a marker of fatigue in such circumstances. The ratios that showed moderate changes under fatigue were TD:iTRIMP and MMP:iTRIMP. There has been research speculating on the notion of pacing in soccer match-play [26]. If pacing is occurring, then the internal load should change in uniform fashion to the external load. Where it doesn’t change in uniform fashion this may be an indication of player’s inability to keep up with the demands of a given scenario and fatigue rather than pacing. Where large game to game variation in external load alone make meaningful inferences about fitness and fatigue difficult, the use of a ratios to assess fitness, fatigue and pacing presents an avenue for research and exploration in practice. Recent research in rugby demonstrating the reliability of some external:internal TL ratio’s in skill and game based activities [27] further enhance the practical applicability of external:internal load ratios. However, to fully understand the utility of the measure for the detection of fatigue in soccer reliability of these measures need to be assessed in a similar way in training activities prominent in soccer. Furthermore, cross-validation with objective markers of neuromuscular fatigue is also required. Although some interesting findings are reported in this paper, the conclusions that can be reached should be tempered and treated with caution due to the small sample size used. The results of this study therefore should be seen as an exploratory starting point rather than a definitive diagnostic tool.

In summary, PL MMP and HP metrics of external load showed similar relationships with fitness when used as a ratio as with previously reported relationships with TD and HID [3] in addressing the first aim of the study. MMP alone may not change in a similar fashion to other external load measures alone, however when used as a ratio it shows similar changes to TD:iTRIMP under fatigue. Ratios not encompassing the whole game (HID/HP) and PL only showed small to trivial changes demonstrating utility in both rested and fatigued conditions for the assessment of fitness but limited usefulness as a proxy for fatigue compared to other ratios. TD:iTRIMP and MMP:iTRIMP showed acute changes that open the avenue for its potential use as a marker of detecting fatigue.

## 5. Conclusions

All measures of external load show large relationships with measures of fitness when used as a ratio, with more complex measures such as MMP or PL not showing added value. When players are fatigued some relationships between ratios and fitness become weaker and, therefore, should be used with caution to infer fitness. However, the acute changes in ratios under fatigue present an avenue for further research on the detection of fatigue especially in sports where a reduction in external output does not necessarily imply fatigue.

## Figures and Tables

**Table 1:** Training load values for both simulations.

Training Load	iTRIMP (AU)		TD (m)		HID (m)		PL (AU)		MMP (W.kg^−1^)		HP (m)	
BEAST_90mod_	1	2	1	2	1	2	1	2	1	2	1	2
Mean	409	304	10,810	10,604	3336	2868	1301	1257	8.74	8.75	3450	3110
SD	174	91	664	592	718	754	94	145	1.01	0.46	691	715
*p* value	0.01		0.17		0.05		0.43		0.98		0.14	
Cohens *d*	−0.79		−0.33		−0.64		−0.37		0.01		−0.48	
90%CI	(−1.27 to −0.37)		(−0.73 to 0.08)		(−1.14 to −0.13)		(−1.19 to 0.45)		(−0.74 to 0.76)		(−1.03 to 0.06)	
Qualitative Descriptor	Large		Small		Moderate		Small		Trivial		Small	
Mechanistic Inference	Most likely −ve		Very likely −ve		Most likely −ve		Unclear		Unclear		Very likely −ve	

**Table 2:** External:Internal ratio values for both simulations.

	TD:iTRIMP		HID:iTRIMP		PL:iTRIMP		MMP:iTRIMP		HP:iTRIMP	
BEAST_90mod_	1	2	1	2	1	2	1	2	1	2
Mean	30.1	37.71	9.65	9.93	3.65	4.50	2.44	3.11	9.92	10.78
SD	10.94	11.26	4.8	3.14	1.49	1.58	0.98	0.93	4.89	3.12
*p* value	<0.01		0.78		0.04		<0.01		0.40	
Cohen’s *d*	0.69		0.07		0.55		0.69		0.21	
90%CI	0.33 to 1.04		−0.39 to 0.53		0.14 to 0.97		0.36 to 1.03		−0.23 to 0.66	
Qualitative Descriptor	Moderate		Trivial		Small		Moderate		Small	
Mechanistic Inference	Very likely +ve		Unclear		Likely +ve		Very likely +ve		Unclear	

**Table 3:** Relationships between ratios and fitness in rested and fatigued states.

	TD:iTRIMP		HID:iTRIMP		PL:iTRIMP		MMP:iTRIMP		HP:iTRIMP	
BEAST_90mod_	1	2	1	2	1	2	1	2	1	2
vLT	0.69	0.6	0.58	0.25	0.64	0.5	0.59	0.57	0.54	0.27
CI	0.22 to 0.9	0.07 to 0.87	0.04 to 0.86	−0.35 to 0.70	0.14 to 0.88	−0.07 to 0.82	0.06 to 0.86	0.03 to 0.85	−0.02to 0.84	−0.33 to 0.72
ES	Large	Large	Large	Small	Large	Moderate	Large	Large	Large	Small
Mechanistic Inference	Very likely +ve	Likely +ve	Likely +ve	Unclear	Very likely +ve	Likely +ve	Likely +ve	Likely +ve	Likely +ve	Unclear
vOBLA	0.58	0.35	0.65	0.74	0.59	0.21	0.61	0.38	0.67	0.70
CI	0.04 to 0.86	−0.25 to 0.76	0.15 to 0.88	0.32 to 0.92	0.05 to 0.86	−0.39 to 0.68	0.09 to 0.87	−0.22 to 0.77	0.19 to 0.89	0.24 to 0.90
ES	Large	Moderate	Large	Very Large	Large	Small	Large	Moderate	Large	Very Large
Mechanistic Inference	Likely +ve	Unclear	Very likely +ve	Very likely +ve	Likely +ve	Unclear	Likely +ve	Unclear	Very likely +ve	Very likely +ve
